# Real-World Effectiveness, Safety, and Tolerability of Facilitated Subcutaneous Immunoglobulin 10% in Secondary Immunodeficiency Disease: A Systematic Literature Review

**DOI:** 10.3390/jcm14041203

**Published:** 2025-02-12

**Authors:** Maria Dimou, Angelo Vacca, Silvia Sánchez-Ramón, Ewa Karakulska-Prystupiuk, Vikte Lionikaite, Csaba Siffel, Colin Anderson-Smits, Marta Kamieniak

**Affiliations:** 1 Department of Hematology and Bone Marrow Transplantation Unit, National and Kapodistrian University of Athens, 15772 Athens, Greece; 2 Unit of Internal Medicine “Guido Baccelli”, Department of Precision and Regenerative Medicine and Ionian Area (DiMePRe-J), University of Bari Aldo Moro, Piazza Giulio Cesare, 11, I-70124 Bari, Italy; 3 Hospital Clínico San Carlos, Complutense University of Madrid, E-28040 Madrid, Spain; 4 Department of Hematology, Transplantation and Internal Medicine, Medical University of Warsaw, 02-091 Warsaw, Poland; 5 Oxford PharmaGenesis, Oxford OX13 5QJ, UK; 6 Takeda Development Center Americas, Inc., Cambridge, MA 02142, USA; 7 College of Allied Health Sciences, Augusta University, Augusta, GA 30912, USA

**Keywords:** secondary immunodeficiency, hyaluronidase-facilitated subcutaneous immunoglobulin (fSCIG) 10%, real-world evidence, infection, safety, tolerability

## Abstract

**Background**: Secondary immunodeficiency disease (SID) is a complex, heterogeneous condition that occurs when extrinsic factors weaken the immune system. Expert consensus guidelines recommend immunoglobulin replacement therapy to manage immunoglobulin G (IgG) levels and mitigate severe, recurrent, and persistent infections. Hyaluronidase-facilitated subcutaneous immunoglobulin (fSCIG) 10% is a dual-vial unit of IgG and recombinant human hyaluronidase; the latter enables absorption of higher volumes of IgG than conventional subcutaneous therapies. **Methods**: For this systematic literature review, Embase, MEDLINE^®^, and the Cochrane Library were searched on 9 August 2023, with supplemental congress searches. **Results**: Eight studies fulfilled the inclusion criteria, reporting real-world evidence of the clinical effectiveness, safety, and tolerability of fSCIG 10% in 183 patients with SID in Europe from September 2014 to August 2021. The potential causes of SID were primarily hematological malignancies, most commonly chronic lymphocytic leukemia. Treatment was typically administered at 4-week or 3-week intervals, with doses of approximately 0.4 g/kg/month. Infections were rare during follow-up, with numerical reductions observed after fSCIG 10% treatment initiation compared with the period before initiation. Adverse reactions, including local infusion site reactions, and tolerability events were uncommon. **Conclusions**: Given the recency of fSCIG 10% use in patients with SID, there are opportunities for future research to better understand survival and patient-reported outcomes after receiving this treatment. Despite SID heterogeneity, this study demonstrates the feasibility of fSCIG 10% treatment for this condition.

## 1. Introduction

Secondary immunodeficiency disease (SID) occurs when the immune system is weakened by extrinsic factors, which include underlying hematological and other chronic diseases and their treatments, certain medications/immunosuppressive agents, solid organ and allogeneic hematopoietic stem cell transplantation (HSCT), and protein loss [1]. The prevalence of SID is increasing and is estimated to be 30 times more common than primary immunodeficiency disease (PID), which has a prevalence of approximately 6 per 10,000 people in the USA [1,2].

The presentation of SID varies widely, depending on the severity of extrinsic factors and the patient’s susceptibility. Patients usually present with immune dysfunctions that can manifest as hypogammaglobulinemia (a quantitatively lower-than-normal immunoglobulin G [IgG] level), as more subtle deficiencies in IgG subclass or functional antibodies, or as insufficient titers of functional antibodies in the presence of a normal total IgG level [3]. Owing to low serum IgG levels, patients are susceptible to severe, recurrent, or persistent infections, and if other immune complications are present, there is a higher risk of end-organ damage and death [4,5]. There are slight differences in the definition of severe infections among the literature. For instance, European expert consensus guidelines define severe infections in patients with hematological malignancies as infections requiring acute intravenous intervention, immediate or prolonged hospitalization, or emergency intensive care treatment [6], whereas American Academy of Allergy, Asthma and Immunology (AAAAI) practical guidance defines severe infections as requiring an emergency department visit or hospitalization, requiring intravenous antibiotics, or reactive antibiotic/antiviral/antifungal therapy specifically for the purposes of treatment [7].

Several guidelines with differing recommendations exist for the diagnosis and management of SID, thereby creating challenges for healthcare professionals [7,8,9,10,11]. Given the heterogeneity of conditions associated with the disease, the approach to management varies according to patient history and the underlying condition [7]. Typically, the underlying condition should be treated, or other causative factors removed if possible, before addressing treatment options for SID. European and Middle East expert consensus Delphi exercises have highlighted that monitoring IgG levels and making therapeutic adjustments using immunoglobulin (Ig) replacement therapy (IgRT) is important for patients with hematological malignancies initiating anti-cancer therapy, and that IgRT should be considered for patients undergoing allogeneic HSCT [6,12]. IgRT is used to mitigate the risk of serious bacterial and some viral infections. However, there is a lack of consensus regarding the use of IgRT in SID [13], which is primarily based on existing clinical experience in treating PID [3,14].

Various types of Ig therapies are available, each with associated benefits and limitations. Conventional subcutaneous Ig (SCIG) provides more stable Ig concentrations with a smaller difference between IgG peaks and troughs than intravenous Ig (IVIG) but it has a lower maximum volume of infusion that necessitates more frequent dosing than IVIG [15,16]. The lack of a need for venous access with conventional SCIG provides a reduced need for clinic visits and greater flexibility for patients; SCIG also has fewer systemic side effects than IVIG [15,16].

Hyaluronidase-facilitated subcutaneous immunoglobulin (fSCIG) 10% (HyQvia; Baxalta Innovations GmbH, a Takeda company, Vienna, Austria) is a dual-vial unit of IgG 10% and recombinant human hyaluronidase (rHuPH20) [17,18]. rHuPH20 transiently increases the permeability of subcutaneous tissue; therefore, fSCIG allows larger volumes of Ig to be administered and absorbed at a single infusion site than conventional SCIG (volumes similar to those with IVIG) [19]. fSCIG 10% can also be administered at a faster rate than conventional SCIG [19]. As with conventional SCIG, fSCIG 10% results in more prolonged Ig absorption into the intravascular space than IVIG and therefore a smaller difference between minimum and maximum IgG concentrations and a longer time to reach peak concentration, which results in more stable IgG levels than IVIG [20]. The bioavailability of fSCIG 10% within the vascular compartment is higher than that of conventional SCIG (92% versus 67% bioavailability, respectively); IVIG has a bioavailability of 100% [19]. fSCIG 10% is approved as a replacement therapy in patients of all ages with PID in Europe and in adults and pediatric patients aged 2–16 years with PID in the USA [17,18]. Additionally, in Europe, fSCIG 10% is indicated for treatment of patients with SID who experience severe or recurrent infections or ineffective antimicrobial treatment and have either proven specific antibody failure or a serum IgG level lower than 4 g/L [17].

The safety and efficacy of fSCIG 10% as a replacement therapy has been well established in clinical trials for patients with PID [20,21,22,23]. US Food and Drug Administration guidance recommends that a serious infection rate less than 1.0 per person-year is adequate to provide substantial evidence of the efficacy of IgRT [24]. Real-world evidence has also demonstrated the long-term effectiveness of fSCIG 10% in PID, as well as flexibility in administration, allowing for an individualized management approach [25,26,27].

Not only is the use of fSCIG 10% well established for patients with PID, real-world studies have also provided insights into its efficacy and safety in patients with SID. Therefore, our aim was to review and summarize all available real-world evidence of the clinical effectiveness, safety, and tolerability of fSCIG 10% in patients with SID, as well as patient-reported and health-related quality of life outcomes, healthcare resource utilization, and administration parameters.

## 2. Methods

This SLR was conducted in accordance with the 2020 Preferred Reporting Items for Systematic Reviews and Meta-Analyses (PRISMA) guidelines, and the study protocol was registered with the International Prospective Register of Systematic Reviews (PROSPERO; registration CRD42023455329).

### 2.1. Eligibility Criteria

To meet the objectives of this SLR, eligible studies included patients (of any age) with SID treated with fSCIG 10% in observational studies to capture data from real-world settings (Supplementary Table S1). Studies involving both patients with SID and PID were included if any outcomes were reported for patients with SID exclusively. In such cases, only data for the subgroup of patients with SID were summarized.

### 2.2. Information Sources

Embase, MEDLINE^®^, and the Cochrane Library were searched on 9 August 2023 using a search string including free-text terms for SID and fSCIG 10% (Supplementary Table S2). The searches were restricted to English language and excluded case reports. Supplemental searches were also conducted to identify relevant publications presented at the following congresses between 1 January 2021 and 9 August 2023: European Myeloma Network annual meeting, International Conference on Malignant Lymphoma, and the Society of Hematologic Oncology annual meeting. The bibliographies of all included articles and relevant narrative/systematic reviews were checked to identify any further relevant articles. To ensure the recency of this study, the search was re-run on 22 July 2024.

### 2.3. Citation Screening

The database search results were deduplicated [28], and two reviewers independently screened article titles and abstracts using Rayyan software (Cambridge, MA, USA, https://help.rayyan.ai/hc/en-us, accessed on 22 July 2024) [29] for compliance with the eligibility criteria (Supplementary Table S1), with any uncertainties resolved by discussion. All irrelevant titles and/or abstracts were excluded.

### 2.4. Full-Text Review and Data Extraction

Full texts of titles and abstracts meeting the eligibility criteria were obtained and screened further for eligibility (Supplementary Table S1) by one reviewer, with uncertainties resolved by discussion with a second reviewer. All relevant data were extracted from studies into a spreadsheet by a single reviewer. All extractions were quality checked by another independent reviewer.

### 2.5. Quality Assessment

For each included study, quality assessment was performed by a single reviewer using the Newcastle–Ottawa Scale for cohort studies [30].

## 3. Results

### 3.1. Overview of the Studies

**Figure 1:**
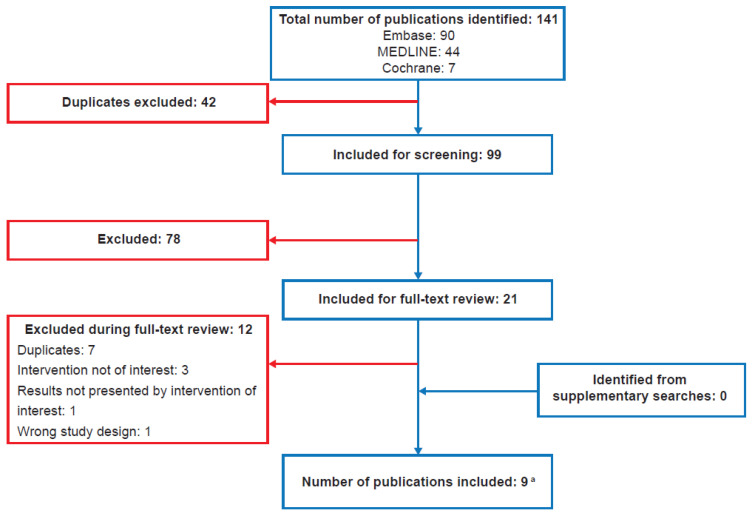
Preferred Reporting Items for Systematic Reviews and Meta-Analyses (PRISMA) flow diagram. ^a^ A full-text research article (Dimou et al., 2023 [33]) replaced the related congress abstract (Dimou et al., 2022 [32]) that was identified during the systematic searches.

Of the 141 publications identified by the electronic searches, 42 were excluded as duplicates and 99 were included for screening (Figure 1). During screening and full-text review, a further 78 and 12 publications were excluded, respectively, resulting in nine publications reporting on eight studies (two publications reported the Facilitated Immunoglobulin Administration Registry and Outcomes [FIGARO] study: one included the combined cohort of patients with PID and SID, and one included only the SID subgroup) [31,32]. No additional articles were identified in supplementary congress searches; however, after the searches were performed, a full-text research article for the FIGARO SID subgroup analysis (Dimou et al., 2023) [33], corresponding to an included congress abstract (Dimou et al., 2022) [32], was published and therefore replaced the congress abstract for data analysis. The resulting publications included seven full-text research articles [31,33,34,35,36,37,38], one congress abstract [39], and one congress poster [40] (Figure 1 and Table 1).

The identified studies reported data from 1574 patients with SID, of whom 183 received fSCIG 10%. All publications were observational in nature, including five retrospective studies (of which two were single-center, two were multicenter, and one used a national database), two prospective studies (one single-center and one multicenter), and one qualitative interview study. The studies reported data collected in the Czech Republic, France, Germany, Greece, Italy, the Netherlands, Poland, Spain, and Sweden from September 2014 to August 2021 (Table 1). The reported average (mean or median) duration of follow-up for patients treated with fSCIG 10% ranged from 6 to 39 months.

### 3.2. Quality of Evidence

The included studies scored 4–6 out of a possible 9 points on the Newcastle–Ottawa Scale relating to the quality of selection and comparability of patients, and the outcome of interest.

### 3.3. Patient Characteristics

Although all eight studies included data for patients with SID, five of the nine articles also reported combined data for patients with PID and for patients with SID; this SLR presents data for patients with SID only unless otherwise stated. Three studies focused on adults (aged ≥18 years) [36,37,38], one of which included only patients aged ≥65 years (the SENEQA study [Study in the Elderly: Non-interventional retrospective HyQvia Assessment]) [38]; one study included only children (aged <18 years) [35]; three included adults and children [31,33,40]; and one did not report the age of the participants [39].

The patient characteristics for each study are presented in Table 1. In studies that reported on the presence of comorbidities, 80–100% of patients had at least one concomitant disease [33,38,40]. Comorbidities included cancer [33,40], cardiovascular disease [33,40], respiratory disorders [31,33,40], gastrointestinal disease [33], and diabetes [40], among others. Mean (standard deviation; SD) duration of SID since diagnosis was reported in two studies as 6.0 (4.8) months (at the time of the first fSCIG 10% infusion [35]) and 10.4 (7.4) months [38]. In a combined cohort of patients with SID and PID, the median (interquartile range; IQR) duration of disease since diagnosis was 40.5 (6.8–89.8) months [34].

Explicit definitions of SID among the included studies were uncommon. The definition in one study of a population of patients treated with SCIG was based on the exclusion of patients with PID-related International Classification of Diseases tenth revision codes [40]. Another study defined SID as secondary hypogammaglobulinemia due to hematologic malignancies with at least two episodes of severe bacterial infection within the previous 12 months [36]. Other studies provided no operationalized methodological definitions.

**Figure 2:**
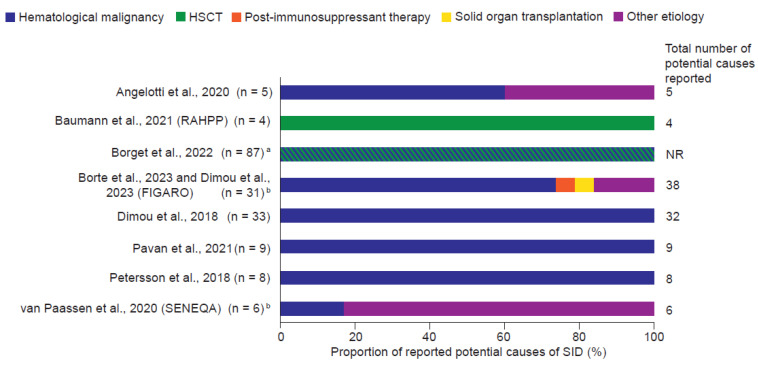
Potential causes of SID [31,33,34,35,36,37,38,39,40]. ^a^ Borget et al. (2022) listed, but did not quantify, the potential causes as “chronic lymphocytic leukemia, multiple myeloma, or patients pre-and post-allogeneic hematopoietic stem-cell transplantation” [40]. ^b^ The FIGARO and SENEQA studies listed these categories as “indications for IgRT”. FIGARO, Facilitated Immunoglobulin Administration Registry And Outcomes; HSCT, hematopoietic stem cell transplantation; IgRT, immunoglobulin replacement therapy; RAHPP; Retrospective chart Analysis of HyQvia usage in Pediatric Patients with PID or SID; SENEQA, Study in the Elderly: Non-interventional retrospective HyQvia Assessment; SID, secondary immunodeficiency disease.

The most common potential cause of SID (Figure 2) was hematological malignancy, mostly reported (in six studies) as chronic lymphocytic leukemia [33,34,36,37,38,40]. Other causes included transplantation (solid organ and both autologous and allogeneic hematopoietic stem cell) [33,35,40] and related therapy (e.g., rituximab) [33], and immunosuppressive treatment for autoimmune disorders [33].

Concomitant medication use was common and varied. Borget et al. (2022) reported that patients received a mean (SD) of 2.0 (1.8) concomitant medications while being treated with fSCIG 10% [40]; the medications received had the following Anatomical Therapeutic Chemical classification system codes: A (alimentary tract and metabolism), H02 (corticosteroids for systemic use), J01 (antibacterials for systemic use), J02 A (antimycotics for systemic use), J07 BB (influenza vaccines), L01 (antineoplastic agents), L03 (immunostimulants), L04 (immunosuppressants), M (musculoskeletal system), P01 (antiprotozoals), and R03 (drugs for obstructive airway diseases) [40]. In the FIGARO study, the most common concomitant therapies reported at fSCIG 10% treatment initiation (in >20% of patients) were *Pneumocystis jirovecii* pneumonia prophylaxis antibiotics (45.2% of patients), viro-statics (41.9%), and antibiotics (25.8%) [33]. In the SENEQA study, 100% of patients were receiving concomitant medications, including supportive therapies such as antibiotics or corticosteroids and antihypertensive agents [38]. Treatments received before fSCIG 10% initiation included other Ig therapies (route of administration not specified) [31,38], IVIG [33,36], SCIG [33], proteasome inhibitors, carfilzomib, immunomodulatory agents, pomalidomide, and daratumumab-based therapy [39].

### 3.4. fSCIG 10% Treatment and Infusion Characteristics

**Figure 3:**
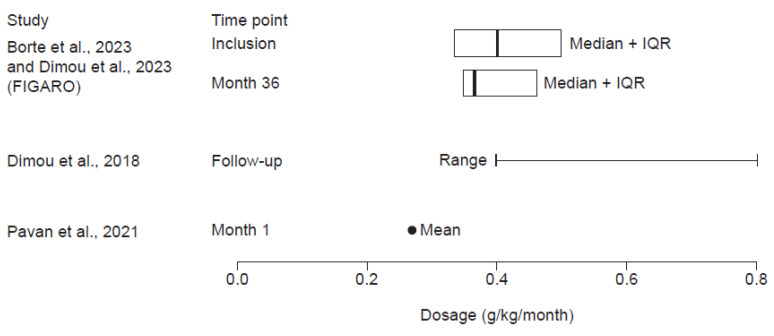
fSCIG 10% doses reported in studies with available data for patients with SID only [31,33,36,39]. FIGARO, Facilitated Immunoglobulin Administration Registry and Outcomes; fSCIG, hyaluronidase-facilitated subcutaneous immunoglobulin; IQR, interquartile range; SID, secondary immunodeficiency disease.

In the FIGARO study, the median monthly dose at inclusion was reported to be 0.40 g/kg at inclusion and 0.37 g/kg at 36 months (Figure 3) [31,33]. Dimou et al. (2018) reported a monthly dose range of 0.4–0.8 g/kg [36], and Pavan et al. (2021) reported a mean dose of 0.27 g/kg [39].

**Figure 4:**
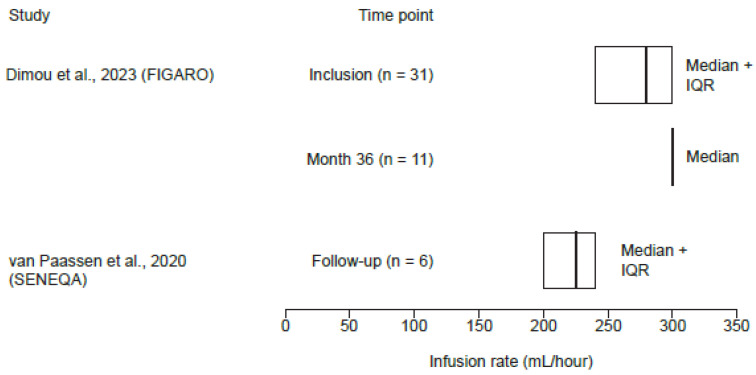
fSCIG 10% infusion rates reported in studies with available data for patients with SID only [33,38]. FIGARO, Facilitated Immunoglobulin Administration Registry and Outcomes; fSCIG, hyaluronidase-facilitated subcutaneous immunoglobulin; IQR, interquartile range; SENEQA, Study in the Elderly: Non-interventional retrospective HyQvia Assessment; SID, secondary immunodeficiency disease.

In the FIGARO study, the median (IQR) maximum infusion rate was 280 (240–300) mL/h at inclusion and 300 (300–300) mL/h at 36 months (Figure 4) [33]. In the SENEQA study, the median (IQR) infusion rate was 225 (200–240) mL/h for single administrations (Figure 4) [38]. Dimou et al. (2018) reported a gradual increase in infusion rate for the first infusion from 10 mL/h to 300 mL/h with increments at 10 min intervals, and if this rate was well tolerated, it was used for all subsequent administrations [36].

The most common dosing interval was every 4 weeks/monthly: 4/6 patients (66.7%) in the SENEQA study [38], 9/11 patients (81.8%) in the FIGARO study at 36 months [33], and all patients in the Borget et al., 2022 study [40]. Three-week dosing intervals were also used by some patients in the SENEQA and FIGARO studies at 36 months: 2/6 (33.3%) patients and 1/11 (9.1%) patients, respectively [33,38]. Dimou et al. (2018) reported that on completion of the fSCIG 10% escalation program (shortened dosing intervals of 1–2 weeks), the dosing interval was 3–4 weeks [36]. Other than for the fSCIG 10% dose ramp-up, use of other dosing frequencies (including 1- and 2-week intervals) was uncommon throughout the studies; one study reported the use of a 2-week interval in just one patient (9.1%) during follow-up [33].

Data from three studies showed that most patients infused into a single site, the most common of which was the abdomen [33,36,38]. Four studies reported that administration during follow-up was more common at home than in a clinical setting [33,36,38,39].

### 3.5. fSCIG 10% Effectiveness

Two studies reported infection rates [31,36]. In the FIGARO study, in the 12 months before inclusion, 32.3% of patients had acute serious bacterial infections (ASBIs), and no ASBIs were reported during the study follow-up period [33]. Six other bacterial infections were reported in 6/25 patients (24%) at 12 months after treatment initiation (bronchitis, n = 2; appendicitis, n = 1; sinusitis, n = 1; urinary tract inflammation, n = 1; paranasal sinus infection, n = 1); at 36-month follow-up, one patient had COVID-19 [33]. Among patients who had 36 months of follow-up, the overall bacterial infection rate was 9.1% (1/11 patients) at 36 months. The low number of patients with 36 months of follow-up was a result of many patients being enrolled later in the fixed study period, rather than participant discontinuations. Only three patients were lost to follow-up [33]. The other study to report infection rates (Dimou et al., 2018) found that 6/33 patients (18.1%) had at least one episode of infection while receiving fSCIG 10%, including lower respiratory tract infection (n = 4), lower extremity nail infection (n = 1), and flu-like infection and dermal infection (*Staphylococcus aureus*) of a lower limb (n = 1) [36]. All six had IgG levels lower than 6 g/L at the time of infection, and the study noted that when the dosing interval was shortened from 4 weeks to 3 weeks in these patients, no new episode of infection was noticed after the dose modification [36].

**Figure 5:**
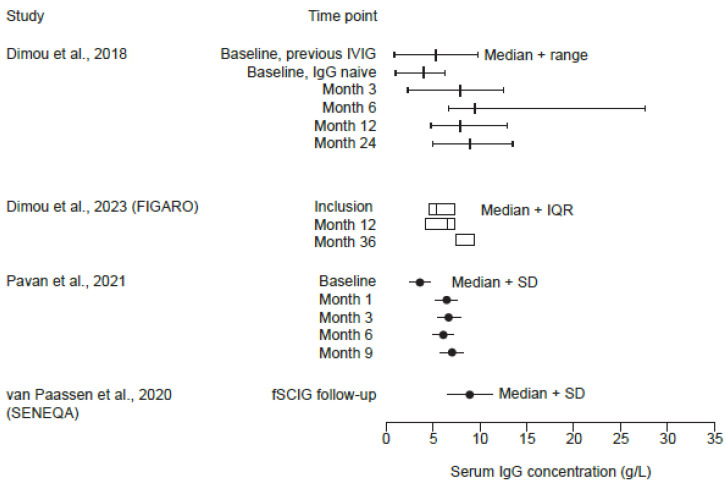
Serum IgG concentrations reported in studies with available data for patients with SID only [33,36,38,39]. FIGARO, Facilitated Immunoglobulin Administration Registry and Outcomes; fSCIG, hyaluronidase-facilitated subcutaneous immunoglobulin; IgG, immunoglobulin G; IQR, interquartile range; IVIG, intravenous immunoglobulin; SD, standard deviation; SENEQA, Study in the Elderly: Non-interventional retrospective HyQvia Assessment.

Four studies included data on serum IgG concentrations (Figure 5) [33,36,38,39]. In three studies that reported baseline values [33,36,39], the average IgG levels were lower than 6 g/L, and during follow-up, exceeded 6 g/L, and there was a trend of increasing serum IgG levels at 24 months and 36 months in the Dimou et al. (2018) [36] and FIGARO [33] studies, respectively. In the SENEQA study, the mean (SD) serum IgG concentration was 9.0 (2.5) g/L, which was interpreted by physicians in the study to be optimal [38].

Although neither survival nor mortality was analyzed in any of the included studies, four deaths were reported in two studies: three due to underlying malignancy [36] and one with unspecified cause [33].

### 3.6. fSCIG 10% Safety and Tolerability

Total and treatment-related adverse reactions were rare across studies, and there were nominal reductions in the number of adverse events during follow-up. One study reported that three patients (9.0%) experienced low-grade fever and headache in the evening after the first and/or second infusions, but not after subsequent infusions [36]. Local edema, which may occur after high-volume subcutaneous administration, was noted in a single study but was mild and resolved within 48 h [36]. In the FIGARO study, 2/31 patients (6.5%) experienced adverse reactions associated with the fSCIG 10% infusion at treatment initiation (one case of infusion site inflammation and one case of headache), with no further adverse reactions reported during follow-up [33]. Pavan et al. (2021) did not observe any infusion-related reactions [39].

Tolerability events were uncommon across all studies with available data. In the FIGARO study, there was one infusion interruption owing to infusion/technical problems at the 36-month follow-up visit [33]. Dimou et al. (2018) reported that one patient presented with unilateral scrotal edema a few hours after their infusions, and the infusions were rescheduled to just before bedtime; however, it is not clear whether any infusions were interrupted [36]. One patient who completed their infusion training refused to start therapy, citing the discomfort caused by subcutaneous post-infusion swelling [34]. Although the SENEQA study did not directly report tolerability events, the physicians reported a 100% positive perception of treatment adherence [38].

### 3.7. Patient-Reported Outcomes, Health-Related Quality of Life, and Healthcare Resource Utilization with fSCIG 10% Treatment

Only one study assessed patient-reported outcomes [37]. Assessment was performed after at least 6 months after switching from another Ig or after initiating with no previous Ig treatment experience [37]. In interviews, eight patients were asked to rate their treatment experience with fSCIG 10% as “better than before”, “neither good nor bad”, or “worse than before” [37]. The majority of patients responded “better than before” regarding efficacy against infections (6/8, 75%), change in their quality of life (7/8, 88%), and satisfaction with the treatment (7/8, 88%) [37]. “Worse than before” was selected by one patient (13%) for the efficacy against infections and by one patient (13%) for change in their quality of life [37]. No studies reported health-related quality of life outcomes.

In studies with available data, infusion training sessions typically lasted 3 weeks [39] or four training sessions [36,38]. The mean (SD) number of nurse visits per month to patients’ homes ranged from 1.3 (2.9) visits to administer fSCIG 10% infusions over 24 months of follow-up [33] to 3.1 (5.1) visits (reasons not specified) over a mean (SD) follow-up of 7.5 (4.6) months [40]. One study reported a range of 2–16 home visits by a nurse in the previous year of fSCIG 10% therapy (retrospective review of medical records) [38].

Hospitalizations for infection or fSCIG 10% administration were rare. Angelotti et al. (2020) reported no hospitalizations for infection [34]. Borget et al. (2022) reported a mean (SD) of 0.5 (0.8) hospitalizations per month for fSCIG 10% administration over the study period (7.5 [4.6] months of follow-up); most of these hospitalizations occurred within the first month of follow-up (1.9 [1.0] hospitalizations per month) [40].

## 4. Discussion

This SLR examined available real-world evidence associated with fSCIG 10% use in 183 patients with SID in Europe. In studies with available data from September 2014 to August 2021, the findings suggest an improvement in outcomes among cohorts of patients with SID initiating fSCIG 10% with no prior Ig therapy experience, and in those switching from other IgRTs. Infections were rare during follow-up periods, and average serum IgG concentrations were elevated after baseline, exceeding 6 g/L during fSCIG 10% treatment and maintained over the course of follow-up. Increased levels of IgG have been shown to be associated with reductions in infections [41], as evidenced by the occurrence of infection only in patients with IgG levels lower than 6 g/L before treatment with fSCIG 10%. It was not possible to determine whether infections reported in the included studies would have been defined as severe according to the European expert consensus or AAAAI guidelines [6,7]. Although infections were rare during follow-up, there was some evidence that, in patients with serum IgG levels lower than 6 g/L at time of infection, shortening the dosing interval from 4 weeks to 3 weeks resulted in eliminating subsequent infections during treatment, supporting fSCIG 10% treatment individualization in patients with SID. Additionally, most infusions were at a single site, with fewer infusion sites used than for conventional SCIG. After infusion training and during follow-up, fSCIG 10% administration at home was common, with most patients self-administering infusions and with few related monthly nurse visits. These findings demonstrate the convenience, flexibility and low healthcare resource utilization of fSCIG 10%. A previous SLR of IVIG and SCIG in patients with SID reported beneficial clinical outcomes and improvements in health-related quality of life [13], suggesting that IgRT, regardless of formulation and administration, may be beneficial in patients with SID. Nonetheless, patient health-related quality of life reports demonstrate preference for SCIG over IVIG owing to reduced adverse effects and hospital visits, and home-based SCIG can offer cost savings versus IVIG [42,43,44]. Furthermore, fSCIG 10% may confer additional benefits in terms of flexibility and convenience from the patient perspective relative to IVIG and SCIG, and it also has fewer systemic side effects than IVIG [16,20].

The dosing strategies in the included studies largely follow the label recommendations. The European Medical Agency fSCIG 10% label recommends that IgG trough level and incidence of infection should be measured, with doses adjusted as necessary to achieve optimal protection against infections [17]. The recommended dose is 0.2–0.4 g/kg every 3–4 weeks, and dose modifications can be considered according to the persistence of infections [17]. With the exception of HSCT recipients, patients with SID are typically of advanced age with a broad range of severe underlying pathologies, many of whom have additional comorbidities and are receiving concomitant medications. The fSCIG 10% summary of product characteristics carries a warning for thromboembolic events, listing advanced age and comorbidities as risk factors [17]; aging, leukemia and its treatment, and advanced age of patients with leukemia have all been associated with venous thromboembolism [45,46,47,48]. With advancing age, the risk of thrombocytopenia and the use of anticoagulant medications increases [49,50]. It is therefore prudent to consider the safety of fSCIG 10% within the context of these factors. A positive fSCIG 10% safety profile has previously been demonstrated in a small study of patients aged 46–60 years with severe PID-related comorbidities such as autoimmune cytopenias [51]. Tolerability has also been demonstrated in two patients with thrombocytopenia and common variable immunodeficiency who did not report any local hemorrhagic manifestations at the infusion site [52]. A study of 47 elderly patients with PID receiving conventional SCIG found no bruising or bleeding despite concomitant anticoagulant treatment [53]. In the present analysis of the studies that reported comorbidities, thrombocytopenia was rare but a broad range of severe conditions were reported; despite this and the SID severity and heterogeneity, infection rates were lower during follow-up than before treatment initiation, adverse events during fSCIG 10% treatment were rare, and fSCIG 10% was well tolerated across the identified studies.

A strength of this study is that only data relating to patients with SID were extracted from studies involving patients with PID as well as patients with SID, thereby maximizing the number of studies that could be included. However, most of the studies reported some data for combined PID/SID cohorts, limiting the data that could be extracted from those studies. Additionally, owing to the recent approval of fSCIG 10% in patients with SID, the searches for inclusion in this analysis returned relatively few studies overall, and data regarding the effectiveness of fSCIG 10% with respect to infections, serum IgG concentrations, and mortality were sparse. Given the focus on real-world evidence, the majority of included studies were retrospective in nature, limiting the ability to infer causal relationships. Differences in statistics and the units for outcomes, as well as uncertainty regarding the definition of SID and the therapeutic threshold of serum IgG concentration considered therapeutic, also made it challenging to directly compare outcomes for analyses. Furthermore, given the heterogeneity in etiology of SID, even though the overall goals of therapy may be similar, the criteria by which to define effectiveness that accurately reflect the real-world outcomes in individual patients may be open to discussion. Although only two studies provided definitions of SID among the patient population, given the real-world use of fSCIG 10% among the studies, it may be that the definition as indicated on the fSCIG 10% label was used. Because the original literature searches were conducted on 9 August 2023, a search was re-run on 22 July 2024 and determined that no new publications during this period were eligible for inclusion.

This analysis identified a number of gaps in the literature. Even though crude mortality reporting was included in two studies, no formal analyses of survival or mortality were available. Additionally, patient-reported outcomes and health-related quality of life were only included in one study using a non-validated measure [37].

### Expert Opinion

The role of IgRT as primary prophylaxis in SID patients remains unclear. Although there are differences among local practices, IgRT is typically prescribed after at least one episode of severe infection and rarely as primary prophylaxis. A clinical decision to prescribe IgRT is largely guided by monitoring IgG levels (at 3 or 6 months) and by considering the patient’s general condition and clinical history, and depends on local IgG product availability. No expert agreement exists on the use of IgRT in patients with hematological malignancies without a history of infections. However, there is consensus on IgRT as primary prophylaxis in patients assessed for hypogammaglobulinemia, namely those receiving chimeric antigen receptor T (CAR T)-cell therapy and bispecific antibody therapies, those with lymphoproliferative disorders or severe comorbidities, and those with IgG levels lower than 4 g/L [12]. CAR T-cell and bispecific antibody therapies have been shown to be highly effective in (often heavily pre-treated) patients with hematological malignancies, enabling these individuals to live longer lives [54,55]. However, these immunotherapies are also associated with significant risk of severe infections and infection-related deaths [54], so it is important to manage the risk of infection proactively in these patients so that they may benefit from improved survival as a result of these effective therapies. Moreover, there is evidence at the time of writing that novel multiple myeloma treatments, such as reversible inhibitors of the chymotrypsin-like activity of the proteasome, affect adaptive immunity [56]. In a randomized control trial, SCIG administration to patients with multiple myeloma reduced the rate of infections, length of hospitalization, and need for antibiotic therapy [57]. Furthermore, SCIG administered concomitantly with anti-tumoral therapy did not induce additive adverse events [57]. Primary prophylaxis with IgRT in patients with SID with hematological malignancies and very low IgG levels could substantially reduce the risk of infections and prevent subsequent organ damage, offering a crucial strategy for preserving long-term health in this vulnerable population. Finally, expert recommendations emphasize the need for more robust randomized controlled trials to evaluate the costs and benefits of primary IgRT prophylaxis [7].

## 5. Conclusions

SID is a complex and heterogeneous condition, with a broad range of potential underlying causes. Despite the heterogeneity of the underlying pathology, comorbidities, and concomitant treatment in patients with SID, this study demonstrates that treatment with fSCIG 10% can be flexible in terms of adjusting the location of care, treatment intervals, and dosing according to individual patient needs, and therefore is feasible in this population.

## Figures and Tables

**Table 1:** Overview of included studies.

Article (Study)	Publication Type	Patients with SID Treated with fSCIG 10%/Study Population with SID, n/N	Country	Study Design	Follow-Up Duration	Patient Characteristics			
						Age, Years, Mean (SD)	Female, n (%)	Race/ Ethnicity, n (%)	Comorbidities, n (%)
Angelotti et al., 2020 [34]	Manuscript	5/5	Italy	Retrospective, observational, monocentric, single-arm, long-term study	Median (IQR), 39 (29–44) months ^a^	NR	NR	NR	NR
Baumann et al., 2021 (RAHPP) [35]	Manuscript	4/4	Germany	Retrospective medical record review	6 months ^a^	8.8 (5.4)	NR	White, 4 (100)	NR
Borget et al., 2022 [40]	Congress poster	87/1478	France	Retrospective cohort from the French National Healthcare database	Mean (SD), 7.5 (4.6) months	68.1 (12.8)	40 (46.0)	NR	Cancer, 73 (83.9) Respiratory disorders, 38 (43.7) Hypertension, 20 (23.0) Cardiovascular disorders, 18 (20.7) Diabetes, 12 (13.8)
Borte et al., 2023 (FIGARO) [31]	Manuscript	31/31	Europe (Czech Republic, Germany, Greece, Italy, Poland, and Spain)	Prospective, observational, multicenter, phase 4 study	Median, 14.5 months ^a^	NR	NR	NR	Splenomegaly, 3 (9.7) Chronic sinusitis, 2 (6.5) Dyslipidemia, 2 (6.5) Anemia, 1 (3.2) Chronic bronchitis, 1 (3.2) Cholecystolithiasis, 1 (3.2) Endometriosis, 1 (3.2) Hypercholesterolemia, 1 (3.2) Leukopenia, 1 (3.2 Lymphadenopathy, 1 (3.2) Lymphopenia, 1 (3.2) Primary biliary cirrhosis, 1 (3.2) Thrombocytopenia, 1 (3.2)
Dimou et al., 2023 (FIGARO) [33] ^b^	Manuscript	31/31	Europe (Czech Republic, Germany, Greece, Italy, Poland, and Spain)	Prospective, observational, multicenter, phase 4 study (SID cohort subgroup analysis)	Maximum, 36 months	61.4 (17.8)	12 (38.7)	White, 29 (93.5)	Arterial hypertension, 13 (41.9) Cancer, 7 (22.6) Gastrointestinal disease, 6 (19.4) Chronic obstructive pulmonary disease, 4 (12.9) Hyperuricemia, 4 (12.9)
Dimou et al., 2018 [36]	Manuscript	33/33	Greece	Single-center retrospective study	Median (range), 11.2 (0.5–27) months	66.1 ^c^	16 (48.5)	NR	NR
Pavan et al., 2021 [39]	Congress abstract	9/9	Italy ^d^	Pilot cohort study	9 months	NR	NR	NR	NR
Petersson et al., 2018 [37]	Manuscript	8/8	Sweden	Interview	NA	NR	NR	NR	NR
van Paassen et al., 2020 (SENEQA) [38]	Manuscript	6/6	Germany and the Netherlands	Retrospective, multicenter medical record review	Median (range), 1.8 (0.1–2.7) years	71.0 (3.2)	4 (67.0)	White, 6 (100)	Any comorbidity, 6 (100)

^a^ Patients with PID and patients with SID combined. ^b^ This full-text research article replaces the related congress abstract (Dimou et al., 2022 [32]) identified during systematic searches. ^c^ Not clear from the source whether the value presented is the mean or the median. ^d^ Country not explicitly stated; assumed from author affiliations. FIGARO, Facilitated Immunoglobulin Administration Registry And Outcomes; fSCIG, hyaluronidase-facilitated subcutaneous immunoglobulin; IQR, interquartile range; NA, not applicable; NR, not reported (for patients with SID only); PID, primary immunodeficiency disease; RAHPP; Retrospective chart Analysis of HyQvia usage in Pediatric Patients with PID or SID; SD, standard deviation; SENEQA, Study in the Elderly: Non-interventional retrospective HyQvia Assessment; SID, secondary immunodeficiency disease.

## Data Availability

Data sharing is not applicable to this article as no data sets were generated or analyzed during the current study.

## Supplementary Materials

The following supporting information can be downloaded at https://www.mdpi.com/article/10.3390/jcm14041203/s1, Supplementary Table S1. Study eligibility criteria, Supplementary Table S2. Search strategy for searches of Embase, MEDLINE, and Cochrane Library performed on 9 August 2023.
