# Supplementary material for: Real-World Effectiveness, Safety, and Tolerability of Facilitated Subcutaneous Immunoglobulin 10% in Secondary Immunodeficiency Disease: A Systematic Literature Review
Source: J Clin Med. 2025 Feb 12;14(4):1203. doi: 10.3390/jcm14041203 (PMC11856383; doi:10.3390/jcm14041203)
Supplement: Supplementary file 1 [file jcm-14-01203-s001.zip › jcm-3276752-supplementary.pdf]

Supplementary Information

Real-World Effectiveness, Safety, and Tolerability of Facilitated Subcutaneous Immunoglobulin 10% in Secondary Immunodeficiency Disease: A Systematic Literature Review

Maria Dimou, Angelo Vacca, Silvia Sánchez-Ramón, Ewa Karakulska-Prystupiuk, Vikte Lionikaite, Csaba Siffel, Colin Anderson-Smits, Marta Kamieniak

Corresponding author: Maria Dimou, Department of Hematology and Bone Marrow Transplantation Unit, National and Kapodistrian University of Athens, 15772 Athens, Greece

Supplementary Table S1. Study eligibility criteria.

Description	Inclusion Criteria	Exclusion Criteria
Population	• Patients with SID<br>• Adults and/or children	• Patients with known PID<br>• Mixed populations where SID outcomes are not reported separately
Intervention	fSCIG 10% (HyQvia)	
Comparators	Any (non-comparator studies will also be considered)	
Outcomes	• Clinical effectiveness, including but not limited to:<br>o serum IgG levels<br>o infection rates (overall and severe, including time to first infection)<br>o survival<br>• Safety/tolerability<br>• PROs/HRQoL<br>• HCRU, including but not limited to:<br>o hospitalizations<br>o antibiotic use<br>o days missed from work/school	• Costs<br>• Economic evaluations
Study design	• Observational (retrospective cohort, case series, cross-sectional and prospective cohort studies, case-control studies)<br>• Reviews (for reference list searching only)	• RCTs/clinical trials<br>• Meta-analyses/indirect treatment comparisons<br>• Surveys<br>• Case reports
Language	English language only	
Country restrictions	None	
Date restrictions	• No time restrictions for full-text publication<br>• Congress abstracts limited to the past 2 years (2021–2023)	

fSCIG, hyaluronidase-facilitated subcutaneous immunoglobulin; HCRU, healthcare resource utilization; HRQoL, health-related quality of life; IgG, immunoglobulin G; PID, primary immunodeficiency disease; PRO, patient reported outcome; RCT, randomized controlled trial; SID, secondary immunodeficiency disease.

Supplementary Table S2. Search strategy for searches of Embase, MEDLINE, and Cochrane Library performed on 9 August 2023.

Category	#	Searches	Embase	MEDLINE	Cochrane
Disease	1	(Secondary immunodeficien* or secondary immuno-deficien* or secondary immune deficien* or sid).mp.	6677	4556	313
Intervention	2	(HyQvia or hyaluronidase*).mp.	13,977	10,221	844
	3	((facilitated immune globulin or facilitate immunoglobulin or immune globulin or immunoglobulin or IG) adj (SC or subcutaneous* or 10%)).mp.	301	138	51
	4	((SC or subcutaneous* or 10%) adj (facilitated immune globulin or facilitated immunoglobulin or immune globulin or immunoglobulin or IG)).mp.	1469	601	140
	5	Human normal immunoglobulin.mp.	137	79	65
	6	or/2-5	15,679	10,986	1066
Disease AND intervention	7	1 and 6	**96**	**43**	**7**

Searches included Embase (1974–8 August 2023), MEDLINE (1946–8 August 2023), and Cochrane Methodology Register, Cochrane Database of Systematic Reviews (2005–2 August 2023), Cochrane Clinical Answers (updated July 2023), and Cochrane Central Register of Controlled Trials (updated July 2023).
